# Supplementary material for: Solvatochromic Sensitivity of BODIPY Probes: A New Tool for Selecting Fluorophores and Polarity Mapping
Source: Int J Mol Sci. 2023 Jan 7;24(2):1217. doi: 10.3390/ijms24021217 (PMC9860957; doi:10.3390/ijms24021217)
Supplement: Supplementary file 1 [file ijms-24-01217-s001.zip › SI.pdf]

Supporting Information

Solvatochromic sensitivity of BODIPY probes: a new tool for selecting fluorophores and polarity mapping

Felix Y. Telegin [1] (ORCID 0000-0002-8626-2583), Viktoria S. Karpova [2] (ORCID 0000-0001-8979-3593), Anna O. Makshanova [3] (ORCID: 0000-0001-5708-2682), Roman G. Astrakhantsev [4] (ORCID 0000-0001-9880-2826), Yuriy S. Marfin [1*] (ORCID 0000-0002-1620-7175)

[1]G.A. Krestov Institute of Solution Chemistry of the RAS, 1, Akademicheskaya str., Ivanovo, 153045, Russia; ymarfin@gmail.com
[2]Ivanovo State University of Chemistry and Technology, 7, Sheremetevsky Ave, Ivanovo 153000, Russia;
[3]Mendeleev University of Chemical Technology of Russia, 9, p. 33, Miusskaya square, Moscow, 125047, Russia;
[4]National Research University Higher School of Economics, 20, Myasnitskaya str., Moscow, 101000, Russia

Phenomenological approach for quantifying the solvatochromism of the fluorescent probes

The relationships between spectral parameters of absorption and emission are often used for the analysis of the solvatochromic effects and estimation of the dipole moments of the fluorophores in the ground and excited state. A general theory for analysis of the solvent effect was presented in [1,2] and reviewed in [3–5] for different variants of the approaches of Lippert-Mataga, Bilot-Kawski, Bakhshiev, Liptay [4,6–8] as well as their similarities in special cases. The general approach based on the assumptions that the polarisability of the molecules is isotropic is expressed in the following way:

$$\nu_{Abs} - \nu_{Em} = m_f\, f(\varepsilon, n) + \Delta^o \qquad (1)$$

$$\nu_{Abs} + \nu_{Em} = -m_\varphi\, \varphi(\varepsilon, n) + \Sigma^o \qquad (2)$$

with the following solvent functions and parameters

$$m_f = \frac{2(\mu_e - \mu_g)^2}{hca^3};\quad m_\varphi = \frac{2(\mu_e^2 - \mu_g^2)}{hca^3} \qquad (3)$$

A detailed theory for solvent effect assuming polarisability of the fluorophore was developed by Liptay [8,9] and Marsh [10] whose results were applied in [11]. In the case of a spherical Onsager cavity, Liptay's equations are expressed in the following way (according to Kawski's notations)

$$f(\varepsilon, n) = \frac{\frac{\varepsilon-1}{2\varepsilon+1} - \frac{n^2-1}{2n^2+1}}{\left(1-\frac{2\alpha}{a^3}\frac{\varepsilon-1}{2\varepsilon+1}\right)\left(1-\frac{2\alpha}{a^3}\frac{n^2-1}{2n^2+1}\right)} \qquad (4)$$

$$\varphi(\varepsilon) = \frac{\frac{\varepsilon-1}{2\varepsilon+1}}{1-\frac{2\alpha}{a^3}\frac{\varepsilon-1}{2\varepsilon+1}} \qquad (5)$$

h is Plank's constant; c is the speed of light; μ_g *and* μ_e are the vectors of dipole moments in the ground and excited states; ε and n are relative dielectric constant and refractive index of solvent; α and a are the mean isotropic polarisability of the solute and the Onsager cavity radius in a homogeneous dielectric, respectively.

A simple semiempirical approach provided in our recent publications [12,13] is useful for gaining new insight into quantifying the solvatochromism of fluorophores in a high range of their polarity sensitivity.

Functions $f(\varepsilon,n)$ and $\varphi(\varepsilon)$ in Eqs (1) and (2) are based on the physicochemical parameters of the solvents determined experimentally. Numerous attempts of exploring the above mentioned theoretical approach expressed by Eqs (1) and (2) helped to discover an approximate linear relationship between spectral functions $(\nu_{Abs}-\nu_{Em})$ and $(\nu_{Abs}+\nu_{Em})$ used for quantifying the solvatochromism of fluorophores. This point has an empirical background based on an assumption regarding the linear relationship between solvent functions $f(\varepsilon,n)$ and $\varphi(\varepsilon)$:

$$f(\varepsilon,n) = P\varphi(\varepsilon) + Q \qquad (6)$$

$$P,Q - const$$

Correlations of Liptay's functions $f(\varepsilon,n)$ and $\varphi(\varepsilon)$ for a dataset of 251 solvents [14] are demonstrating linear regression coefficient P approximately equal to 1.02 (R^2=0.94-0.98) invariant to changes of polarisability α/a^3 in the range 0-0.5. Therefore, the regression coefficient P in Eq (7) could be approximated by unity, i.e. $P\approx1$.

Correlations of Bilot-Kawski functions $f(\varepsilon,n)$ and $\varphi(\varepsilon,n)$ [1,2] exhibit much worse linear correlation for the total dataset [14], i.e. R^2=0.78, although in case of limited sets of solvents applied in certain studies higher correlations could be found.

A combination of Eqs (1), (2), and (6) yields a desirable linear relationship (7) between absorption and emission wavenumbers of fluorophore:

$$\nu_{Abs} - \nu_{Em} = -P\frac{m_f}{m_\varphi}(\nu_{Abs}+\nu_{Em}) + \left(P\Sigma^0\frac{m_f}{m_\varphi} + Qm_f + \Delta^0\right) \qquad (7)$$

where expressions $P\frac{m_f}{m_\varphi}$ and $\left(P\Sigma^0\frac{m_f}{m_\varphi} + Qm_f + \Delta^0\right)$ are the constants.

The above Eq (7) for the Stokes shift $\nu_{Abs} - \nu_{Em}$ as well as correspondent equations for absorption and emission wavenumbers ν_{Abs} and ν_{Em} could be written shortly:

$$\nu_{Abs} - \nu_{Em} = -A(\nu_{Abs} + \nu_{Em}) + B \quad (8)$$

$$\nu_{Abs} = \frac{1-A}{2}(\nu_{Abs} + \nu_{Em}) + \frac{B}{2} \quad (9)$$

$$\nu_{Em} = \frac{1+A}{2}(\nu_{Abs} + \nu_{Em}) - \frac{B}{2} \quad (10)$$

$$A = \frac{m_f}{m_\varphi} = \frac{(\mu_e - \mu_g)^2}{\mu_e^2 - \mu_g^2} \quad (11)$$

$$B = \Sigma^0 \frac{m_f}{m_\varphi} + Q m_f + \Delta^0 \quad (12)$$

Noteworthy that expression (11) is written in a more general form than in the previous research [12,13]. It helps to use dipole moments in vector form with no additional assumptions because the square of a vector function has a scalar value.

Table S1. List of dyes selected for the dataset

1998 Lopez-Arbeloa-PM567 [15]
1999 Lopez-Arbeloa(177)-PM546 [16]
2004 Banuelos Prieto(29)-PAr1Ac [17]
2004 Banuelos-Prieto(5503)-PM597 [18]
2004 Lopez-Arbeloa-PM650 [19]
2004 Shen-3d [20]
2004 Shen-4a [20]
2004 Shen-4b [20]
2004 Shen-4c [20]
2004 Shen-4e [20]
2004 Shen-4f [20]
2006 Baruah-1 [21]
2006 Qin(190)-1 [22]
2008 Qin-1 [23]
2009 Cieslik-Boczula-2CN [24]
2009 Cieslik-Boczula-4CN [24]
2009 Qin(11731)-1 [25]
2010 Chaudhuri-1b [26]
2010 Filarowski-1 [27]
2010 Filarowski-2 [27]
2010 Filarowski-3 [27]
2010 Leen(2016)-1 [28]
2010 Leen(2016)-2 [28]
2010 Leen(2016)-3 [28]
2011 Banuelos(3437)-BTAA [29]
2012 Boens(9621)-1 [30]
2012 Boens(9621)-2 [30]
2012 Boens(9621)-3 [30]
2012 Boens(9621)-4 [30]
2012 Boens(9621)-5 [30]
2012 Boens(9621)-6 [30]
2012 Yin-1 [31]
2012 Zhao-OH [32]
2013 Er-BDC-9 [33]
2013 Nano-TX(6) [34]
2013 Yang-TPA-BDP1 [35]
2013 Yang-TPA-BDP2 [35]
2013 Yang-TPA-BDP3 [35]
2014 Boens-10 [36]
2014 Boens-6 [36]
2014 Boens-8 [36]
2015 Caltagirone-Py-BODIPY [37]
2015 Feng-1 [38]
2015 Filarowski-A [39]
2015 Jiao-3 [40]
2015 Jiao-4 [40]
2015 Jiao -5 [40]
2015 Jiao-1 [40]
2015 Jiao-2 [40]
2015 Thorat-Dye 2 [41]
2015 Thorat-Dye 3 [41]
2015 Thorat-Dye 4 [41]
2015 Waddell-1 [42]
2015 Waddell-2 [42]
2015 Waddell-3 [42]
2015 Waddell-4 [42]

2015 Waddell-5 [42]
2015 Waddell-6 [42]
2016 Bacalum-1 [43]
2016 Gupta-1 [44]
2016 Marfin(1975)-2 [45]
2016 Marfin(1975)-3 [45]
2016 Marfin(1975)-4 [45]
2016 Marfin(1975)-5 [45]
2016 Orte-2-Ethyn [46]
2016 Orte-2-Ph [46]
2016 Orte-3-Ethyn [46]
2016 Orte-3-Ph [46]
2016 Orte-3-Styryl [46]
2016 Orte-8-Ethyn [46]
2016 Orte-8-Ph [46]
2016 Qin-1 [47]
2016 Telore-7 [48]
2016 Telore-7a [48]
2016 Vu-2 [49]
2016 Vu-3 [49]
2016 Zhu(35627)-BP-2 [50]
2017 Petrushenko(488)-1 [51]
2017 Sadak-15 [52]
2017 Suhina-1 [53]
2017 Thorat-Dye 1 [54]
2017 Thorat-Dye 2 [54]
2017 Zhang(2447)-Ph-TMBDP [55]
2018 Leen-BODIPY [56]
2018 Mallah-Bn-OH-BDY [57]
2018 Ordonez-Hernandez-mVP1 [58]
2018 Ordonez-Hernandez-mVP2 [58]
2018 Ordonez-Hernandez-mVP3 [58]
2018 Ripoll-2 [59]
2018 Ripoll-4 [59]
2018 Ripoll-6 [59]
2019 Ali-10 [60]
2019 Ali-12 [60]
2019 Ali-14 [60]
2019 Ali-16 [60]
2019 Ali-19 [60]
2019 Ali-2 [60]
2019 Ali-20 [60]
2019 Ali-21 [60]
2019 Ali-23 [60]
2019 Ali-6 [60]
2019 Ali-9 [60]
2019 Antina-2,2-CH2-bis(BODIPY) [61]
2019 Antina-2,3-CH2-bis(BODIPY) [61]
2019 Antina-3,3-CH2-bis(BODIPY) [61]
2019 Bai-NJ1060 [62]
2019 Guseva-BODIPY 1 [63]
2019 Kawakami-BFBODIPY-DMP-DMAS [64]
2019 Sevinc-TPy-BDP [65]
2019 Zhang(148)-BDP [66]
2020 Gonzalez-Vera-2 [67]
2020 Gonzalez-Vera-3 [67]
2020 Shen-BODIPY-DT [68]
2021 Vysnauskas-BODIPY2 [69]
2021 Vysnauskas-BODIPY3 [69]

Table S2. List of dyes selected for comparison of duplicates

1999 Lopez Arbeloa(315)-PM546 [70]
1999 Lopez Arbeloa(315)-PM567 [70]
2002 Costela-PM567 [71]
2007 Rohand-2 [72]
2008 Costela-PM567 [73]
2009 Arroyo-1 [74]
2009 Costela-PM597 [75]
2011 Banuelos(677)-BDP [76]
2011 Banuelos(7261)-8-PAB [77]
2011 Banuelos(7261)-BDP [77]
2012 Duran-Sampedro-15 [78]
2012 Osorio-Martinez-1 [79]
2012 Zhang(11215)-1a [80]
2013 Duran-Sampedro-PM546 [81]
2013 Duran-Sampedro-PM567 [81]
2013 Duran-Sampedro-PM650 [81]
2013 Esnal(2691)-9 [82]
2013 Esnal(4134)-3 [83]
2013 Flores-Rizo-BODIPY [84]
2013 Flores-Rizo-BODIPY 7 [84]
2014 Choi-2 [85]
2014 Liu(5471)-B1 [86]
2018 Filatov(8016)-13 [87]
2018 Prasannan-Phenyl-BODIPY [88]
2018 Zhang(13)-BDP [89]
2019 Belmonte-Vazquez-22 [90]
2019 Hu(139)-1 [91]
2019 Hu(15944)-1 [92]
2019 Mallah(122)-Bn-BDY [93]
2019 Mallah(126)-Bn-BPY [94]
2019 Mallah(126)-Bn-OH-BDY [94]
2019 Zhang(286)-BDP [95]
2020 Berezin-3 [96]

Table S3. Solvents used in solvatochromic studies and their frequency of occurance in a dataset of 115 BODIPYs

Solvent, IUPAC name / Occurrence in a dataset		Solvent, IUPAC name / Occurrence in a dataset	
acetonitrile	104	water	7
methanol	100	pyridine	7
ethyl acetate	99	2-methylpropan-2-ol	6
oxolane	92	1-methylpyrrolidin-2-one	6
toluene	88	2-methylbutane	6
trichloromethane	86	2-(propan-2-yloxy)propane	6
1,4-dioxane	77	heptane	5
propan-2-one	74	1-(2,2-dimethylpropoxy)-2,2-dimethylpropane	5
dichloromethane	68	1,4-xylene	4
methanesulfinylmethane	67	2,2,4-trimethylpentane	4
ethoxyethane	66	methyl formate	4
cyclohexane	66	methyl acetate	4
N,N-dimethylformamide	60	2-methoxy-2-methylpropane	3
ethanol	57	1,1,1-trichloroethane	3
butan-1-ol	53	phenylmethanol	3
propan-2-ol	47	cyclohexanol	3
hexane	43	2-methylpropan-1-ol	3
1-butoxybutane	42	butan-2-one	3
octan-1-ol	40	2-chloroethan-1-ol	3
pentan-1-ol	30	pentan-2-one	3
tetrachloromethane	27	propane-1,2,3-triol	2
chlorobenzene	27	2-phenoxyethan-1-ol	2
cyclohexanone	22	undecan-1-ol	2
butanenitrile	21	benzonitrile	1
propan-1-ol	18	2-methyloxolane	1
benzene	15	3-methylbutan-2-one	1
ethane-1,2-diol	14	1,2-dibromoethane	1
decan-1-ol	14	1,2-dichlorobenzene	1
hexan-1-ol	12	2-methoxyethan-1-ol	1
1,2-dichloroethane	12	2-methylheptane	1
butyl acetate	11	pyrrolidin-2-one	1
pentane	9	formamide	1
propanenitrile	8	N-methylformamide	1
acetic acid	8	dimethylarsinate	1
2,2,2-trifluoroethan-1-ol	8	2-[(2-hydroxyethyl)amino]ethan-1-ol	1
methylcyclohexane	7		

References

1. Kawski, A.; Kukliński, B.; Bojarski, P.; Diehl, H. Ground and Excited State Dipole Moments of LAURDAN Determined from Solvatochromic and Thermochromic Shifts of Absorption and Fluorescence Spectra. *Zeitschrift für Naturforschung* **2000**, *55a*, 817–822, doi:10.1515/zna-2000-9-1011.
2. Kawski, A. On the Estimation of Excited-State Dipole Moments from Solvatochromic Shifts of Absorption and Fluorescence Spectra. *Zeitschrift für Naturforschung* **2002**, *57a*, 255–262, doi:10.1515/zna-2002-0509.
3. Minkin, V.I.; Osipov, O.A.; Zhdanov, I.A. *Dipole moments in organic chemistry;* Plenum Press: New York-London, 1970.
4. Reichardt, C.; Welton, T. *Solvents and solvent effects in organic chemistry*, 4th, updated and enl. ed.; Wiley-VCH: Weinheim Germany, 2011, ISBN 3527324739.
5. Varhese, A.; Akshaya, K.B. Application of Fluorescence in Solvatochromic Studies of Organic Compounds. In *Reviews in Fluorescence 2017*; Geddes, C.D., Ed.; Springer International Publishing: Cham, 2018; pp 99–121, ISBN 978-3-030-01568-8.
6. Lakowicz, J.R. *Principles of fluorescence spectroscopy*, 3rd ed.; Springer: New York, 2006, ISBN 0387312781.
7. Bakhshiev, N.G.; Knyazhanskii, M.I.; Minkin, V.I.; Osipov, O.A.; Saidov, G.V. Experimental Determination of the Dipole Moments of Organic Molecules in Excited Electronic Statesf. *Russian Chemical Reviews* **1969**, *38*, 740–754.
8. Liptay, W. Die Lösungsmittelabhängigkeit der Wellenzahl von Elektronenbanden und die chemisch-physikalischen Grundlagen. *A Journal of Physical Sciences: Zeitschrift für Naturforschung A* **1965**, *20*, doi:10.1515/zna-1965-1109.
9. Liptay, W. Dipole Moments and Polarizabílítíes of Molecules In Excited Electronic States. In *Excited states. Volume 1 Volume 1*; Lim, E.C., Ed., 1974; pp 129–229, ISBN 9781483217673 1483217671.
10. Marsh, D. Reaction Fields in the Environment of Fluorescent Probes: Polarity Profiles in Membranes. *Biophysical Journal* **2009**, *96*, 2549–2558, doi:10.1016/j.bpj.2009.01.006.
11. Randles, E.G.; Bergethon, P.R. Reaction Field Analysis and Lipid Bilayer Location for Lipophilic Fluorophores. *J. Phys. Chem. B* **2013**, *117*, 10193–10202, doi:10.1021/jp402861x.
12. Telegin, F.Y.; Marfin, Y.S. New insights into quantifying the solvatochromism of BODIPY based fluorescent probes. *Spectrochim. Acta A Mol. Biomol. Spectrosc.* **2021**, *255*, 119683, doi:10.1016/j.saa.2021.119683.
13. Telegin, F.Y.; Marfin, Y.S. Polarity and Structure of BODIPYs: A Semiempirical and Chemoinformation Analysis. *Russian Journal of Inorganic Chemistry* **2022**, *67*, 362–374, doi:10.1134/S0036023622030135.
14. Cerón-Carrasco, J.P.; Jacquemin, D.; Laurence, C.; Planchat, A.; Reichardt, C.; Sraïdi, K. Solvent polarity scales: determination of new ET (30) values for 84 organic solvents. *J. Phys. Org. Chem.* **2014**, *27*, 512–518, doi:10.1002/poc.3293.
15. López Arbeloa, F.; López Arbeloa, T.; López Arbeloa, I.; García-Moreno, I.; Costela, A.; Sastre, R.; Amat-Guerri, F. Photophysical and lasing properties of pyrromethene567 dye in liquid solution: Environment effects. *Chemical Physics* **1998**, *236*, 331–341, doi:10.1016/S0301-0104(98)00215-8.
16. López Arbeloa, F.; López Arbeloa, T.; López Arbeloa, I. Electronic spectroscopy of pyrromethene 546. *Journal of Photochemistry and Photobiology A: Chemistry* **1999**, *121*, 177–182, doi:10.1016/S1010-6030(98)00453-5.
17. Bañuelos Prieto, J.; López Arbeloa, F.; Martinez Martinez, V.; Arbeloa López, T.; Amat-Guerri, F.; Liras, M.; López Arbeloa, I. Photophysical properties of a new 8-phenyl analogue of the laser dye

PM567 in different solvents: internal conversion mechanisms. *Chemical Physics Letters* **2004**, *385*, 29–35, doi:10.1016/j.cplett.2003.12.076.
18. Bañuelos Prieto, J.; López Arbeloa, F.; Martínez Martínez, V.; Arbeloa López, T.; López Arbeloa, I. Photophysical Properties of the Pyrromethene 597 Dye: Solvent Effect. *J. Phys. Chem. A* **2004**, *108*, 5503–5508, doi:10.1021/jp0373898.
19. López Arbeloa, F.; Bañuelos Prieto, J.; Martínez Martínez, V.; Arbeloa López, T.; López Arbeloa, I. Intramolecular charge transfer in pyrromethene laser dyes: photophysical behaviour of PM650. *Chemphyschem* **2004**, *5*, 1762–1771, doi:10.1002/cphc.200400242.
20. Shen, Z.; Röhr, H.; Rurack, K.; Uno, H.; Spieles, M.; Schulz, B.; Reck, G.; Ono, N. Boron-diindomethene (BDI) dyes and their tetrahydrobicyclo precursors--en route to a new class of highly emissive fluorophores for the red spectral range. *Chemistry* **2004**, *10*, 4853–4871, doi:10.1002/chem.200400173.
21. Baruah, M.; Qin, W.; Flors, C.; Hofkens, J.; Vallée, R.A.L.; Beljonne, D.; van der Auweraer, M.; Borggraeve, W.M. de; Boens, N. Solvent and pH dependent fluorescent properties of a dimethylaminostyryl borondipyrromethene dye in solution. *J. Phys. Chem. A* **2006**, *110*, 5998–6009, doi:10.1021/jp054878u.
22. Qin, W.; Baruah, M.; Borggraeve, W.M. de; Boens, N. Photophysical properties of an on/off fluorescent pH indicator excitable with visible light based on a borondipyrromethene-linked phenol. *Journal of Photochemistry and Photobiology A: Chemistry* **2006**, *183*, 190–197, doi:10.1016/j.jphotochem.2006.03.015.
23. Qin, W.; Baruah, M.; Sliwa, M.; van der Auweraer, M.; Borggraeve, W.M. de; Beljonne, D.; van Averbeke, B.; Boens, N. Ratiometric, fluorescent BODIPY dye with aza crown ether functionality: synthesis, solvatochromism, and metal ion complex formation. *J. Phys. Chem. A* **2008**, *112*, 6104–6114, doi:10.1021/jp800261v.
24. Cieślik-Boczula, K.; Burgess, K.; Li, L.; Nguyen, B.; Pandey, L.; Borggraeve, W.M. de; van der Auweraer, M.; Boens, N. Photophysics and stability of cyano-substituted boradiazaindacene dyes. *Photochem. Photobiol. Sci.* **2009**, *8*, 1006–1015, doi:10.1039/b905054j.
25. Qin, W.; Leen, V.; Dehaen, W.; Cui, J.; Xu, C.; Tang, X.; Liu, W.; Rohand, T.; Beljonne, D.; van Averbeke, B.; et al. 3,5-Dianilino Substituted Difluoroboron Dipyrromethene: Synthesis, Spectroscopy, Photophysics, Crystal Structure, Electrochemistry, and Quantum-Chemical Calculations †. *J. Phys. Chem. C* **2009**, *113*, 11731–11740, doi:10.1021/jp9017822.
26. Chaudhuri, T.; Mula, S.; Chattopadhyay, S.; Banerjee, M. Photophysical properties of the 8-phenyl analogue of PM567: a theoretical rationalization. *Spectrochim. Acta A Mol. Biomol. Spectrosc.* **2010**, *75*, 739–744, doi:10.1016/j.saa.2009.11.048.
27. Filarowski, A.; Kluba, M.; Cieślik-Boczula, K.; Koll, A.; Kochel, A.; Pandey, L.; Borggraeve, W.M. de; van der Auweraer, M.; Catalán, J.; Boens, N. Generalized solvent scales as a tool for investigating solvent dependence of spectroscopic and kinetic parameters. Application to fluorescent BODIPY dyes. *Photochem. Photobiol. Sci.* **2010**, *9*, 996–1008, doi:10.1039/c0pp00035c.
28. Leen, V.; Qin, W.; Yang, W.; Cui, J.; Xu, C.; Tang, X.; Liu, W.; Robeyns, K.; van Meervelt, L.; Beljonne, D.; et al. Synthesis, spectroscopy, crystal structure determination, and quantum chemical calculations of BODIPY dyes with increasing conformational restriction and concomitant red-shifted visible absorption and fluorescence spectra. *Chem. Asian J.* **2010**, *5*, 2016–2026, doi:10.1002/asia.201000248.
29. Bañuelos, J.; Arbeloa, F.L.; Martinez, V.; Liras, M.; Costela, A.; Moreno, I.G.; Arbeloa, I.L. Difluoro-boron-triaza-anthracene: a laser dye in the blue region. Theoretical simulation of alternative difluoro-boron-diaza-aromatic systems. *Phys. Chem. Chem. Phys.* **2011**, *13*, 3437–3445, doi:10.1039/c0cp01147a.

30. Boens, N.; Leen, V.; Dehaen, W.; Wang, L.; Robeyns, K.; Qin, W.; Tang, X.; Beljonne, D.; Tonnelé, C.; Paredes, J.M.; et al. Visible absorption and fluorescence spectroscopy of conformationally constrained, annulated BODIPY dyes. *J. Phys. Chem. A* **2012**, *116*, 9621–9631, doi:10.1021/jp305551w.
31. Yin, Z.; Tam, A.Y.-Y.; Wong, K.M.-C.; Tao, C.-H.; Li, B.; Poon, C.-T.; Wu, L.; Yam, V.W.-W. Functionalized BODIPY with various sensory units--a versatile colorimetric and luminescent probe for pH and ions. *Dalton Trans.* **2012**, *41*, 11340–11350, doi:10.1039/c2dt30446e.
32. Zhao, C.; Feng, P.; Cao, J.; Zhang, Y.; Wang, X.; Yang, Y.; Zhang, Y.; Zhang, J. 6-Hydroxyindole-based borondipyrromethene: Synthesis and spectroscopic studies. *Org. Biomol. Chem.* **2012**, *10*, 267–272, doi:10.1039/c1ob06200j.
33. Er, J.C.; Tang, M.K.; Chia, C.G.; Liew, H.; Vendrell, M.; Chang, Y.-T. MegaStokes BODIPY-triazoles as environmentally sensitive turn-on fluorescent dyes. *Chem. Sci.* **2013**, *4*, 2168, doi:10.1039/c3sc22166k.
34. Nano, A.; Ziessel, R.; Stachelek, P.; Harriman, A. Charge-recombination fluorescence from push-pull electronic systems constructed around amino-substituted styryl-BODIPY dyes. *Chemistry* **2013**, *19*, 13528–13537, doi:10.1002/chem.201301045.
35. Yang, Y.; Zhang, L.; Li, B.; Zhang, L.; Liu, X. Triphenylamine-cored tetramethyl-BODIPY dyes: synthesis, photophysics and lasing properties in organic media. *RSC Adv.* **2013**, *3*, 14993, doi:10.1039/c3ra42276c.
36. Boens, N.; Wang, L.; Leen, V.; Yuan, P.; Verbelen, B.; Dehaen, W.; van der Auweraer, M.; Borggraeve, W.D. de; van Meervelt, L.; Jacobs, J.; et al. 8-HaloBODIPYs and their 8-(C, N, O, S) substituted analogues: Solvent Dependent UV–Vis Spectroscopy, Variable Temperature NMR, Crystal Structure Determination, and Quantum Chemical Calculations. *J. Phys. Chem. A* **2014**, *118*, 1576–1594, doi:10.1021/jp412132y.
37. Caltagirone, C.; Arca, M.; Falchi, A.M.; Lippolis, V.; Meli, V.; Monduzzi, M.; Nylander, T.; Rosa, A.; Schmidt, J.; Talmon, Y.; et al. Solvatochromic fluorescent BODIPY derivative as imaging agent in camptothecin loaded hexosomes for possible theranostic applications. *RSC Adv.* **2015**, *5*, 23443–23449, doi:10.1039/C5RA01025J.
38. Feng, H.-T.; Xiong, J.-B.; Zheng, Y.-S.; Pan, B.; Zhang, C.; Wang, L.; Xie, Y. Multicolor Emissions by the Synergism of Intra/Intermolecular Slipped π–π Stackings of Tetraphenylethylene-DiBODIPY Conjugate. *Chem. Mater.* **2015**, *27*, 7812–7819, doi:10.1021/acs.chemmater.5b03765.
39. Filarowski, A.; Lopatkova, M.; Lipkowski, P.; van der Auweraer, M.; Leen, V.; Dehaen, W. Solvatochromism of BODIPY-Schiff dye. *J. Phys. Chem. B* **2015**, *119*, 2576–2584, doi:10.1021/jp508718d.
40. Jiao, L.; Yu, C.; Wang, J.; Briggs, E.A.; Besley, N.A.; Robinson, D.; Ruedas-Rama, M.J.; Orte, A.; Crovetto, L.; Talavera, E.M.; et al. Unusual spectroscopic and photophysical properties of meso-tert-butylBODIPY in comparison to related alkylated BODIPY dyes. *RSC Adv.* **2015**, *5*, 89375–89388, doi:10.1039/C5RA17419H.
41. Thorat, K.G.; Kamble, P.; Mallah, R.; Ray, A.K.; Sekar, N. Congeners of Pyrromethene-567 Dye: Perspectives from Synthesis, Photophysics, Photostability, Laser, and TD-DFT Theory. *J. Org. Chem.* **2015**, *80*, 6152–6164, doi:10.1021/acs.joc.5b00654.
42. Waddell, P.G.; Liu, X.; Zhao, T.; Cole, J.M. Rationalizing the photophysical properties of BODIPY laser dyes via aromaticity and electron-donor-based structural perturbations. *Dyes and Pigments* **2015**, *116*, 74–81, doi:10.1016/j.dyepig.2015.01.010.
43. Bacalum, M.; Wang, L.; Boodts, S.; Yuan, P.; Leen, V.; Smisdom, N.; Fron, E.; Knippenberg, S.; Fabre, G.; Trouillas, P.; et al. A Blue-Light-Emitting BODIPY Probe for Lipid Membranes. *Langmuir* **2016**, *32*, 3495–3505, doi:10.1021/acs.langmuir.6b00478.

44. Gupta, N.; Reja, S.I.; Bhalla, V.; Gupta, M.; Kaur, G.; Kumar, M. A bodipy based fluorescent probe for evaluating and identifying cancer, normal and apoptotic C6 cells on the basis of changes in intracellular viscosity. *Journal of Materials Chemistry B* **2016**, *4*, 1968–1977, doi:10.1039/C5TB02476E.
45. Marfin, Y.S.; Vodyanova, O.S.; Merkushev, D.A.; Usoltsev, S.D.; Kurzin, V.O.; Rumyantsev, E.V. Effect of π-Extended Substituents on Photophysical Properties of BODIPY Dyes in Solutions. *J. Fluoresc.* **2016**, *26*, 1975–1985, doi:10.1007/s10895-016-1891-3.
46. Orte, A.; Debroye, E.; Ruedas-Rama, M.J.; Garcia-Fernandez, E.; Robinson, D.; Crovetto, L.; Talavera, E.M.; Alvarez-Pez, J.M.; Leen, V.; Verbelen, B.; et al. Effect of the substitution position (2, 3 or 8) on the spectroscopic and photophysical properties of BODIPY dyes with a phenyl, styryl or phenylethynyl group. *RSC Adv.* **2016**, *6*, 102899–102913, doi:10.1039/C6RA22340K.
47. Qin, W.; Dou, W.; Leen, V.; Dehaen, W.; van der Auweraer, M.; Boens, N. A ratiometric, fluorescent BODIPY-based probe for transition and heavy metal ions. *RSC Adv.* **2016**, *6*, 7806–7816, doi:10.1039/C5RA23751C.
48. Telore, R.D.; Jadhav, A.G.; Sekar, N. NLOphoric and solid state emissive BODIPY dyes containing N - phenylcarbazole core at meso position – Synthesis, photophysical properties of and DFT studies. *Journal of Luminescence* **2016**, *179*, 420–428, doi:10.1016/j.jlumin.2016.07.035.
49. Vu, T.T.; Méallet-Renault, R.; Clavier, G.; Trofimov, B.A.; Kuimova, M.K. Tuning BODIPY molecular rotors into the red: sensitivity to viscosity vs. temperature. *J. Mater. Chem. C* **2016**, *4*, 2828–2833, doi:10.1039/C5TC02954F.
50. Zhu, H.; Fan, J.; Mu, H.; Zhu, T.; Zhang, Z.; Du, J.; Peng, X. d-PET-controlled "off-on" Polarity-sensitive Probes for Reporting Local Hydrophilicity within Lysosomes. *Sci. Rep.* **2016**, *6*, 35627, doi:10.1038/srep35627.
51. Petrushenko, K.B.; Petrushenko, I.K.; Petrova, O.V.; Sobenina, L.N.; Trofimov, B.A. Novel environment-sensitive 8-CF3 -BODIPY dye with 4-(dimethylamino)phenyl group at the 3-position: Synthesis and optical properties. *Dyes and Pigments* **2017**, *136*, 488–495, doi:10.1016/j.dyepig.2016.09.009.
52. Sadak, A.E.; Gören, A.C.; Bozdemir, Ö.A.; Saraçoğlu, N. Synthesis of Novel meso- Indole- and meso-Triazatruxene-BODIPY Dyes. *ChemistrySelect* **2017**, *2*, 10512–10516, doi:10.1002/slct.201701897.
53. Suhina, T.; Amirjalayer, S.; Woutersen, S.; Bonn, D.; Brouwer, A.M. Ultrafast dynamics and solvent-dependent deactivation kinetics of BODIPY molecular rotors. *Phys. Chem. Chem. Phys.* **2017**, *19*, 19998–20007, doi:10.1039/c7cp02037f.
54. Thorat, K.G.; Ray, A.K.; Sekar, N. Modulating TICT to ICT characteristics of acid switchable red emitting boradiazaindacene chromophores: Perspectives from synthesis, photophysical, hyperpolarizability and TD-DFT studies. *Dyes and Pigments* **2017**, *136*, 321–334, doi:10.1016/j.dyepig.2016.08.049.
55. Zhang, X.-F.; Feng, N. Photoinduced Electron Transfer-based Halogen-free Photosensitizers: Covalent meso-Aryl (Phenyl, Naphthyl, Anthryl, and Pyrenyl) as Electron Donors to Effectively Induce the Formation of the Excited Triplet State and Singlet Oxygen for BODIPY Compounds. *Chem. Asian J.* **2017**, *12*, 2447–2456, doi:10.1002/asia.201700794.
56. Leen, V.; Laine, M.; Ngongo, J.M.; Lipkowski, P.; Verbelen, B.; Kochel, A.; Dehaen, W.; van der Auweraer, M.; Nadtochenko, V.; Filarowski, A. Impact of the Keto-Enol Tautomeric Equilibrium on the BODIPY Chromophore. *J. Phys. Chem. A* **2018**, *122*, 5955–5961, doi:10.1021/acs.jpca.8b03489.
57. Mallah, R.; Sreenath, M.C.; Chitrambalam, S.; Joe, I.H.; Sekar, N. Excitation energy transfer processes in BODIPY based donor-acceptor system - Synthesis, photophysics, NLO and DFT study. *Optical Materials* **2018**, *84*, 795–806, doi:10.1016/j.optmat.2018.08.007.

58. Ordóñez-Hernández, J.; Jiménez-Sánchez, A.; García-Ortega, H.; Sánchez-Puig, N.; Flores-Álamo, M.; Santillan, R.; Farfán, N. A series of dual-responsive Coumarin-Bodipy probes for local microviscosity monitoring. *Dyes and Pigments* **2018**, *157*, 305–313, doi:10.1016/j.dyepig.2018.05.009.
59. Ripoll, C.; Cheng, C.; Garcia-Fernandez, E.; Li, J.; Orte, A.; Do, H.; Jiao, L.; Robinson, D.; Crovetto, L.; González-Vera, J.A.; et al. Synthesis and Spectroscopy of Benzylamine-Substituted BODIPYs for Bioimaging. *Eur. J. Org. Chem.* **2018**, 2561–2571, doi:10.1002/ejoc.201800083.
60. Ali, H.; Guérin, B.; van Lier, J.E. gem-Dibromovinyl boron dipyrrins: synthesis, spectral properties and crystal structures. *Dalton Transactions* **2019**, *48*, 11492–11507, doi:10.1039/c9dt02309g.
61. Antina, L.A.; Ksenofontov, A.A.; Kalyagin, A.A.; Antina, E.V.; Berezin, M.B.; Khodov, I.A. Luminescent properties of new 2,2-, 2,3- and 3,3-CH2-bis(BODIPY)s dyes: Structural and solvation effects. *Spectrochim. Acta A Mol. Biomol. Spectrosc.* **2019**, *218*, 308–319, doi:10.1016/j.saa.2019.03.117.
62. Bai, L.; Sun, P.; Liu, Y.; Zhang, H.; Hu, W.; Zhang, W.; Liu, Z.; Fan, Q.; Li, L.; Huang, W. Novel aza-BODIPY based small molecular NIR-II fluorophores for in vivo imaging. *Chem. Commun. (Camb)* **2019**, *55*, 10920–10923, doi:10.1039/c9cc03378e.
63. Guseva, G.B.; Ksenofontov, A.A.; Antina, E.V.; Berezin, M.B.; Vyugin, A.I. Effect of solvent nature on spectral properties of blue-emitting meso-propargylamino-BODIPY. *Journal of Molecular Liquids* **2019**, *285*, 194–203, doi:10.1016/j.molliq.2019.04.058.
64. Kawakami, J.; Sasaki, Y.; Yanase, K.; Ito, S. Benzo-fused BODIPY Derivative as a Fluorescent Chemosensor for Fe^{3+}, Cu^{2+}, and Al^{3+}. *Trans. Mat. Res. Soc. Japan* **2019**, *44*, 69–73, doi:10.14723/tmrsj.44.69.
65. Sevinç, G.; Özgür, M.; Küçüköz, B.; Karatay, A.; Aslan, H.; Yılmaz, H. Synthesis and spectroscopic properties of a novel “turn off” fluorescent probe: Thienyl-pyridine substituted BODIPY. *Journal of Luminescence* **2019**, *211*, 334–340, doi:10.1016/j.jlumin.2019.03.058.
66. Zhang, X.-F.; Zhu, J. BODIPY parent compound: Fluorescence, singlet oxygen formation and properties revealed by DFT calculations. *Journal of Luminescence* **2019**, *205*, 148–157, doi:10.1016/j.jlumin.2018.09.017.
67. González-Vera, J.A.; Lv, F.; Escudero, D.; Orte, A.; Guo, X.; Hao, E.; Talavera-Rodriguez, E.M.; Jiao, L.; Boens, N.; Ruedas-Rama, M.J. Unusual spectroscopic and photophysical properties of solvatochromic BODIPY analogues of Prodan. *Dyes and Pigments* **2020**, 108510, doi:10.1016/j.dyepig.2020.108510.
68. Shen, F.; Wang, T.; Yu, X.; Li, Y. Free radical oxidation reaction for selectively solvatochromic sensors with dynamic sensing ability. *Chinese Chemical Letters* **2020**, *31*, 1919–1922, doi:10.1016/j.cclet.2019.12.025.
69. Vyšniauskas, A.; Cornell, B.; Sherin, P.S.; Maleckaitė, K.; Kubánková, M.; Izquierdo, M.A.; Vu, T.T.; Volkova, Y.A.; Budynina, E.M.; Molteni, C.; et al. Cyclopropyl Substituents Transform the Viscosity-Sensitive BODIPY Molecular Rotor into a Temperature Sensor. *ACS Sens.* **2021**, *6*, 2158–2167, doi:10.1021/acssensors.0c02275.
70. López Arbeloa, T.; López Arbeloa, F.; López Arbeloa, I.; García-Moreno, I.; Costela, A.; Sastre, R.; Amat-Guerri, F. Correlations between photophysics and lasing properties of dipyrromethene–BF2 dyes in solution. *Chemical Physics Letters* **1999**, *299*, 315–321, doi:10.1016/S0009-2614(98)01281-0.
71. Costela, A.; García-Moreno, I.; Gomez, C.; Sastre, R.; Amat-Guerri, F.; Liras, M.; López Arbeloa, F.; Bañuelos Prieto, J.; López Arbeloa, I. Photophysical and Lasing Properties of New Analogs of the Boron−Dipyrromethene Laser Dye PM567 in Liquid Solution. *J. Phys. Chem. A* **2002**, *106*, 7736–7742, doi:10.1021/jp0209897.
72. Rohand, T.; Lycoops, J.; Smout, S.; Braeken, E.; Sliwa, M.; van der Auweraer, M.; Dehaen, W.; Borggraeve, W.M. de; Boens, N. Photophysics of 3,5-diphenoxy substituted BODIPY dyes in solution. *Photochem. Photobiol. Sci.* **2007**, *6*, 1061–1066, doi:10.1039/b705921c.

73. Costela, A.; García-Moreno, I.; Pintado-Sierra, M.; Amat-Guerri, F.; Liras, M.; Sastre, R.; Arbeloa, F.L.; Bañuelos Prieto, J.; Arbeloa, I.L. New laser dye based on the 3-styryl analog of the BODIPY dye PM567. *Journal of Photochemistry and Photobiology A: Chemistry* **2008**, *198*, 192–199, doi:10.1016/j.jphotochem.2008.03.010.
74. Arroyo, I.J.; Hu, R.; Merino, G.; Tang, B.Z.; Peña-Cabrera, E. The smallest and one of the brightest. Efficient preparation and optical description of the parent borondipyrromethene system. *J. Org. Chem.* **2009**, *74*, 5719–5722, doi:10.1021/jo901014w.
75. Costela, A.; García-Moreno, I.; Pintado-Sierra, M.; Amat-Guerri, F.; Sastre, R.; Liras, M.; López Arbeloa, F.; Bañuelos Prieto, J.; López Arbeloa, I. New analogues of the BODIPY dye PM597: photophysical and lasing properties in liquid solutions and in solid polymeric matrices. *J. Phys. Chem. A* **2009**, *113*, 8118–8124, doi:10.1021/jp902734m.
76. Bañuelos, J.; Arroyo-Córdoba, I.J.; Valois-Escamilla, I.; Alvarez-Hernández, A.; Peña-Cabrera, E.; Hu, R.; Zhong Tang, B.; Esnal, I.; Martínez, V.; López Arbeloa, I. Modulation of the photophysical properties of BODIPY dyes by substitution at their meso position. *RSC Adv.* **2011**, *1*, 677, doi:10.1039/c1ra00020a.
77. Bañuelos, J.; Martín, V.; Gómez-Durán, C.F.A.; Arroyo Córdoba, I.J.; Peña-Cabrera, E.; García-Moreno, I.; Costela, Á.; Pérez-Ojeda, M.E.; Arbeloa, T.; López Arbeloa, I. New 8-amino-BODIPY derivatives: surpassing laser dyes at blue-edge wavelengths. *Chemistry – A European Journal* **2011**, *17*, 7261–7270, doi:10.1002/chem.201003689.
78. Duran-Sampedro, G.; Agarrabeitia, A.R.; Garcia-Moreno, I.; Costela, A.; Bañuelos, J.; Arbeloa, T.; López Arbeloa, I.; Chiara, J.L.; Ortiz, M.J. Chlorinated BODIPYs: Surprisingly Efficient and Highly Photostable Laser Dyes. *Eur. J. Org. Chem.* **2012**, *2012*, 6335–6350, doi:10.1002/ejoc.201200946.
79. Osorio-Martínez, C.A.; Urías-Benavides, A.; Gómez-Durán, C.F.A.; Bañuelos, J.; Esnal, I.; López Arbeloa, I.; Peña-Cabrera, E. 8-AminoBODIPYs: cyanines or hemicyanines? The effect of the coplanarity of the amino group on their optical properties. *J. Org. Chem.* **2012**, *77*, 5434–5438, doi:10.1021/jo300724m.
80. Zhang, M.; Hao, E.; Xu, Y.; Zhang, S.; Zhu, H.; Wang, Q.; Yu, C.; Jiao, L. One-pot efficient synthesis of pyrrolylBODIPY dyes from pyrrole and acyl chloride. *RSC Adv.* **2012**, *2*, 11215, doi:10.1039/c2ra22203e.
81. Durán-Sampedro, G.; Agarrabeitia, A.R.; Cerdán, L.; Pérez-Ojeda, M.E.; Costela, A.; García-Moreno, I.; Esnal, I.; Bañuelos, J.; Arbeloa, I.L.; Ortiz, M.J. Carboxylates versus Fluorines: Boosting the Emission Properties of Commercial BODIPYs in Liquid and Solid Media. *Adv. Funct. Mater.* **2013**, *23*, 4195–4205, doi:10.1002/adfm.201300198.
82. Esnal, I.; Urías-Benavides, A.; Gómez-Durán, C.F.A.; Osorio-Martínez, C.A.; García-Moreno, I.; Costela, A.; Bañuelos, J.; Epelde, N.; López Arbeloa, I.; Hu, R.; et al. Reaction of amines with 8-methylthioBODIPY: dramatic optical and laser response to amine substitution. *Chem. Asian J.* **2013**, *8*, 2691–2700, doi:10.1002/asia.201300760.
83. Esnal, I.; Valois-Escamilla, I.; Gómez-Durán, C.F.A.; Urías-Benavides, A.; Betancourt-Mendiola, M.L.; López-Arbeloa, I.; Bañuelos, J.; García-Moreno, I.; Costela, A.; Peña-Cabrera, E. Blue-to-orange color-tunable laser emission from tailored boron-dipyrromethene dyes. *Chemphyschem* **2013**, *14*, 4134–4142, doi:10.1002/cphc.201300818.
84. Flores-Rizo, J.O.; Esnal, I.; Osorio-Martínez, C.A.; Gómez-Durán, C.F.A.; Bañuelos, J.; López Arbeloa, I.; Pannell, K.H.; Metta-Magaña, A.J.; Peña-Cabrera, E. 8-Alkoxy- and 8-aryloxy-BODIPYs: straightforward fluorescent tagging of alcohols and phenols. *J. Org. Chem.* **2013**, *78*, 5867–5877, doi:10.1021/jo400417h.
85. Choi, S.; Bouffard, J.; Kim, Y. Aggregation-induced emission enhancement of a meso-trifluoromethyl BODIPY via J-aggregation. *Chem. Sci.* **2014**, *5*, 751–755, doi:10.1039/C3SC52495G.

86. Liu, C.-L.; Chen, Y.; Shelar, D.P.; Li, C.; Cheng, G.; Fu, W.-F. Bodipy dyes bearing oligo(ethylene glycol) groups on the meso-phenyl ring: tuneable solid-state photoluminescence and highly efficient OLEDs. *Journal of Materials Chemistry C* **2014**, *2*, 5471, doi:10.1039/c4tc00720d.
87. Filatov, M.A.; Karuthedath, S.; Polestshuk, P.M.; Callaghan, S.; Flanagan, K.J.; Telitchko, M.; Wiesner, T.; Laquai, F.; Senge, M.O. Control of triplet state generation in heavy atom-free BODIPY-anthracene dyads by media polarity and structural factors. *Phys. Chem. Chem. Phys.* **2018**, *20*, 8016–8031, doi:10.1039/c7cp08472b.
88. Prasannan, D.; Arunkumar, C. A "turn-on-and-off" pH sensitive BODIPY fluorescent probe for imaging E. coli cells. *New J. Chem.* **2018**, *42*, 3473–3482, doi:10.1039/C7NJ04313A.
89. Zhang, X.-F.; Feng, N. Attaching naphthalene derivatives onto BODIPY for generating excited triplet state and singlet oxygen: Tuning PET-based photosensitizer by electron donors. *Spectrochim. Acta A Mol. Biomol. Spectrosc.* **2018**, *189*, 13–21, doi:10.1016/j.saa.2017.08.005.
90. Belmonte-Vázquez, J.L.; Avellanal-Zaballa, E.; Enríquez-Palacios, E.; Cerdán, L.; Esnal, I.; Bañuelos, J.; Villegas-Gómez, C.; López Arbeloa, I.; Peña-Cabrera, E. Synthetic Approach to Readily Accessible Benzofuran-Fused Borondipyrromethenes as Red-Emitting Laser Dyes. *J. Org. Chem.* **2019**, *84*, 2523–2541, doi:10.1021/acs.joc.8b02933.
91. Hu, W.; Lin, Y.; Zhang, X.-F.; Feng, M.; Zhao, S.; Zhang, J. Heavy-atom-free charge transfer photosensitizers: Tuning the efficiency of BODIPY in singlet oxygen generation via intramolecular electron donor-acceptor interaction. *Dyes and Pigments* **2019**, *164*, 139–147, doi:10.1016/j.dyepig.2019.01.019.
92. Hu, W.; Liu, M.; Zhang, X.-F.; Wang, Y.; Wang, Y.; Lan, H.; Zhao, H. Can BODIPY-Electron Acceptor Conjugates Act As Heavy Atom-Free Excited Triplet State and Singlet Oxygen Photosensitizers via Photoinduced Charge Separation-Charge Recombination Mechanism? *J. Phys. Chem. C* **2019**, *123*, 15944–15955, doi:10.1021/acs.jpcc.9b02961.
93. Mallah, R.R.; Mohbiya, D.R.; Sreenath, M.C.; Chitrambalam, S.; Joe, I.H.; Sekar, N. NLOphoric benzyl substituted BODIPY and BOPHY: A comprehensive linear and nonlinear optical study by spectroscopic, DFT and Z-scan measurement. *Spectrochim. Acta A Mol. Biomol. Spectrosc.* **2019**, *215*, 122–129, doi:10.1016/j.saa.2019.02.097.
94. Mallah, R.R.; Mohbiya, D.R.; Sreenath, M.C.; Chitrambalam, S.; Joe, I.H.; Sekar, N. Non-linear optical response of meso hybrid BODIPY: Synthesis, photophysical, DFT and Z scan study. *Spectrochim. Acta A Mol. Biomol. Spectrosc.* **2019**, *209*, 126–140, doi:10.1016/j.saa.2018.10.040.
95. Zhang, X.-F.; Zhu, J. BODIPY parent compound: Excited triplet state and singlet oxygen formation exhibit strong molecular oxygen enhancing effect. *Journal of Luminescence* **2019**, *212*, 286–292, doi:10.1016/j.jlumin.2019.04.050.
96. Berezin, M.B.; Antina, E.V.; Guseva, G.B.; Kritskaya, A.; Semeikin, A.S. Effect of meso-phenyl substitution on spectral properties, photo- and thermal stability of boron (III) and zinc (II) dipyrrometenates. *Inorganic Chemistry Communications* **2020**, *111*, 107611, doi:10.1016/j.inoche.2019.107611.
